# Engineering macrophages to phagocytose cancer cells by blocking the CD47/SIRPɑ axis

**DOI:** 10.1002/cam4.2332

**Published:** 2019-06-11

**Authors:** Hongcheng Yang, Ruoyang Shao, Hongxin Huang, Xinlong Wang, Zhili Rong, Ying Lin

**Affiliations:** ^1^ Cancer Research Institute School of Basic Medical Sciences, Southern Medical University Guangzhou China; ^2^ Dermatology Hospital Southern Medical University Guangzhou China

**Keywords:** cancer, CD47, immunotherapy, macrophages, SIRPɑ

## Abstract

The use of immunotherapy has achieved great advances in the treatment of cancer. Macrophages play a pivotal role in the immune defense system, serving both as phagocytes (removal of pathogens and cancer cells) and as antigen‐presenting cells (activation of T cells). However, research regarding tumor immunotherapy is mainly focused on the adaptive immune system. The usefulness of innate immune cells (eg, macrophages) in the treatment of cancer has not been extensively investigated. Recent advances in synthetic biology and the increasing understanding of the cluster of differentiation 47/signal regulatory protein alpha (CD47/SIRPɑ) axis may provide new opportunities for the clinical application of engineered macrophages. The CD47/SIRPɑ axis is a major known pathway, repressing phagocytosis and activation of macrophages. In this article, we summarize the currently available evidence regarding the CD47/SIRPɑ axis, and immunotherapies based on blockage. In addition, we propose cell therapy strategies based on macrophage engineering.

## 1 INTRODUCTION

The immune system, including innate and adaptive immune cells, plays important roles in the maintenance of homeostasis and prevention of carcinogenesis. Cancer immunotherapy has demonstrated impressive efficacy in the treatment of certain previously “incurable” cancers, leading a new approach in tumor research and treatment. Numerous attempts focus on the activation of adaptive immune cells, especially T cells. These include immune checkpoint blockade, exemplified by anti‐cytotoxic T‐lymphocyte‐associated protein 4 (anti‐CTLA‐4), anti‐programmed death‐ligand 1 (anti‐PD‐1) and anti‐PD‐L1 antibodies, and chimeric antigen receptor (CAR) T‐cell therapy.^1^, ^2^, ^3^ Innate immune cells constitute the first line of immune response. Nevertheless, at present, few cancer immunotherapies focus on these cells. Considering their potent phagocytosis and antigen presentation capability, macrophages may be engineered to treat cancers. However, tumor‐associated macrophages often manifest a pro‐tumorigenic effect. The cluster of differentiation 47/signal regulatory protein alpha (CD47/SIRPɑ) axis plays a critical role in inhibiting the activation of macrophages against cancer. Blockage of the CD47/SIRPɑ axis is a successful strategy to stimulate macrophages against both hematologic and solid malignancies.^4^ In this review, we will discuss the strategies of macrophage engineering to achieve an anti‐tumor effect through blockage of the CD47/SIRPɑ axis.

## 2 CD47/SIRPⱭ AXIS SIGNAL

**Figure 1:**
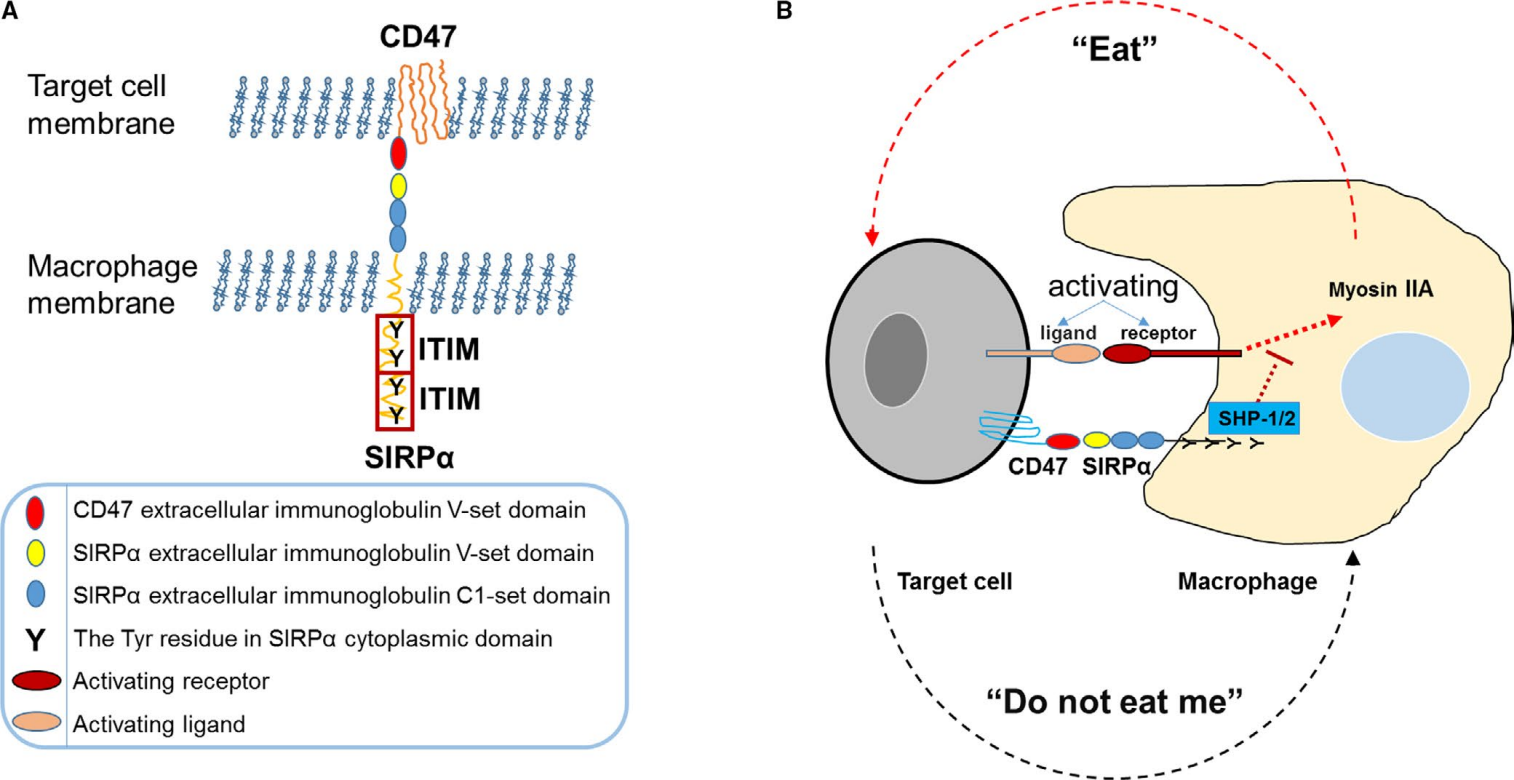
The cluster of differentiation 47/signal regulatory protein alpha (CD47/SIRPα) axis is an inhibitory signal for macrophages. (A) The schematic structures of CD47 and SIRPα. The extracellular region of SIRPα contains 3 Ig‐like domains, including an NH_2_‐terminal V‐set domain and two C1‐set domains. There are 4 Tyr residues in the cytoplasmic domain that form two typical inhibitory immunoreceptor tyrosine‐based inhibitory motifs (ITIMs). Of note, the extracellular region of CD47 contains an Ig‐like domain that can bind to the SIRPα NH_2_‐terminal V‐set domain. (B) The “Eat” and “Do not eat me” signals in macrophages. Phagocytosis in macrophages is regulated through both activation and inhibition of receptor signals. Following stimulation by their ligands, the activating receptors of macrophages send a phagocytic signal that induces the “eat” process. After the binding of SIRPα—the inhibiting receptor—to CD47 on target cells, the cytoplasmic tail is phosphorylated, leading to the recruitment and activation of the protein tyrosine phosphatases SHP‐1 and SHP‐2. Through currently uncharacterized mechanisms, these two phosphatases ultimately result in the suppression of the function of myosin IIA, which activates phagocytosis

The transmembrane protein CD47 is widely and variably expressed in all types of cells. In contrast, the expression of SIRPɑ is restricted to macrophages, granulocytes, monocytes, dendritic cells, and neurons with varied levels.^5^, ^6^ CD47 contains 1 immunoglobulin‐like (Ig‐like) domain in the extracellular region and 5 transmembrane domains. SIRPɑ contains 3 Ig‐like domains in the extracellular region, including 1 NH_2_‐terminal V‐set domain, and 2 C1‐set domains.^7^ The intracellular region of SIRPɑ contains 2 typical immunoreceptor tyrosine‐based inhibitory motifs (ITIMs) that function as inhibitory signal initiators (Figure 1A). The NH_2_‐terminal V‐set domain of SIRPɑ recognizes the Ig‐like domain of CD47. The interaction between SIRPα and CD47 may promote the phosphorylation of SIRPα ITIMs that induce the recruitment and activation of protein tyrosine phosphatases SHP‐1 and SHP‐2. These phosphatases lead to the dephosphorylation of downstream molecules and ultimately, the repression of phagocytosis^8^ (Figure 1B). In macrophages, one of the potential mechanisms involved in this inhibitory cascade is the suppression of myosin IIA that is critical for phagocytosis.^9^ Thus, in the absence of CD47 binding to SIRPɑ, lack of the ITIM inhibitory signal cascade allows the activation of receptors to initiate phagocytosis (Figure 1B).

In 2000, CD47 was shown to be a self‐marker for red blood cells (RBCs), which interacts with SIRPɑ to inhibit red pulp macrophage phagocytosis. This finding demonstrated that the CD47/SIRPɑ axis is essential for RBC maintenance.^10^ The expression of CD47 is also upregulated in circulating hematopoietic stem cells and progenitor cells.^11^ In addition, the non‐obese diabetic‐severe combined immunodeficiency (NOD‐SCID) mouse strain was shown to be an outstanding recipient model for the engraftment of human hematopoietic cells. This was attributed to the high affinity exhibited by the polymorphic SIRPɑ for human CD47 in a NOD background. The interaction between human CD47 and mouse SIRPɑ inhibits the ability of NOD mouse macrophages to attack human grafts.^12^

## 3 THE CD47/SIRPⱭ AXIS IN CANCER

**Figure 2:**
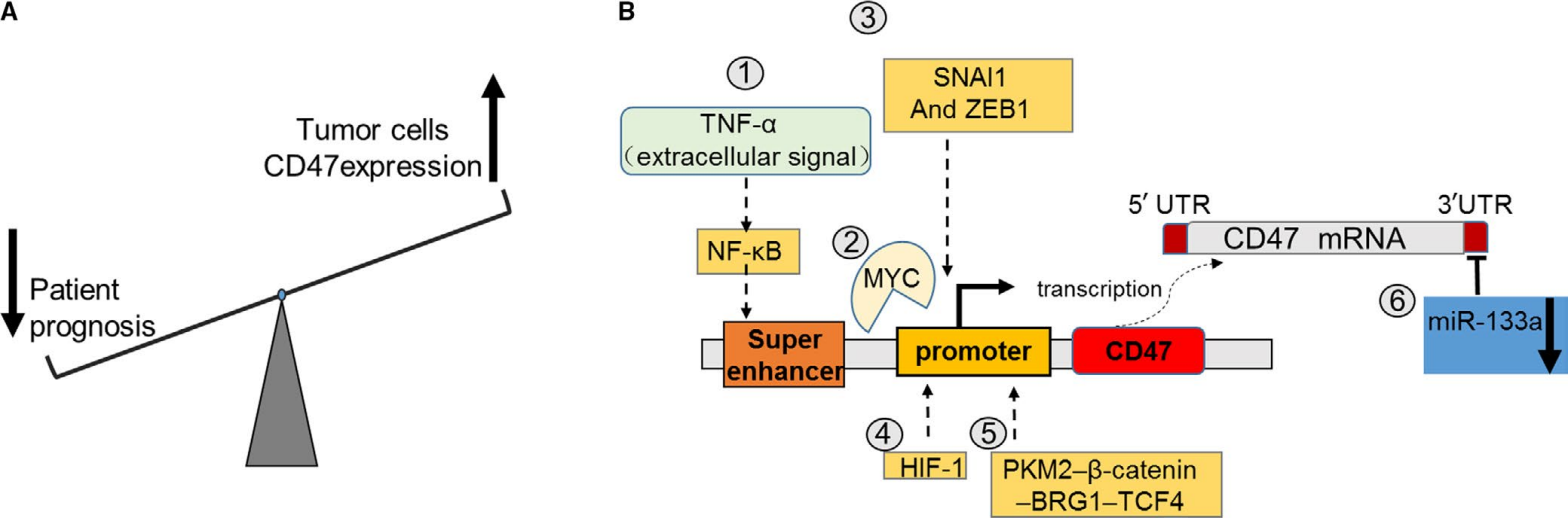
The expression of CD47 in cancer cells. (A) A high expression level of CD47 is correlated to poor prognosis in cancers. (B) Outline of the currently known mechanisms inducing the overexpression of CD47 in cancer cells. Following activation by extracellular TNF‐α, NF‐κB binds to a super‐enhancer to promote the expression of CD47. Transcription factors enhance the expression of CD47 by directly binding to the promoter of CD47. The expression of tumor suppressor miR‐133a is downregulated in cancers, leading to the decreased expression of CD47 by binding to the 3 prime untranslated region of CD47 mRNA

The CD47/SIRPɑ axis functions as a protective signal to prevent the clearance of hematopoietic cells by macrophages under physiological conditions. Under pathological conditions, cancer cells may hijack the axis to avoid immune surveillance. In fact, CD47 is overexpressed in various kinds of cancer cells, and its high expression level is positively correlated to poor prognosis (Figure 2A). The expression level of CD47 is higher in acute myeloid leukemia (AML) stem cells vs their normal counterparts. The expression of CD47 is also associated with poor prognosis in adult AML patients.^13^ In solid tumors, it was also found that the expression level of CD47 was higher than that observed in the surrounding normal tissues.^14^ The high level of CD47 expression correlates with worse prognosis in ovarian cancer, gliomas, squamous cell carcinoma of the head and neck, melanoma, and osteosarcoma.^14^, ^15^, ^16^, ^17^, ^18^ There are various mechanisms related to the upregulated expression of CD47 in different tumors, including the binding of NF‐κB transcription factor to a CD47‐associated super‐enhancer,^19^ as well as direct binding of MYC,^20^ SNAI1 And ZEB1,^21^ HIF‐1,^22^ and the PKM2–β‐catenin–BRG1‐TCF4 complex^23^ to the promoter of CD47 (Figure 2B). As an established oncogene, MYC binds to the promoter of both CD47 and PD‐L1 in cells of solid and hematologic malignancies.^20^ In addition, microRNAs may contribute to the overexpression of CD47 in cancers. MiR‐133a is a tumor suppressor gene, and its expression is invariably downregulated in cancers. It has been reported that miR‐133a may bind to the 3 prime untranslated region of CD47 mRNA, and contribute to the overexpression of CD47 in cancers.^24^ In numerous human‐derived xenograft murine models,^25^, ^26^, ^27^ blockage of the CD47/SIRPɑ axis using an antibody has been found to stimulate phagocytosis of cancer cells by macrophages in vitro and inhibit tumor growth in vivo. Therapies involving the blockage of the CD47/SIRPɑ axis also showed efficacy in xenograft models transplanted with patient‐derived AML stem cells.^13^ In conclusion, the binding of CD47 to SIRPα presents a negative signal to immune cells, especially macrophages, and promotes the survival of tumor cells.

## 4 TREATMENT OF CANCERS BY BLOCKING THE CD47/SIRPⱭ AXIS

**Figure 3:**
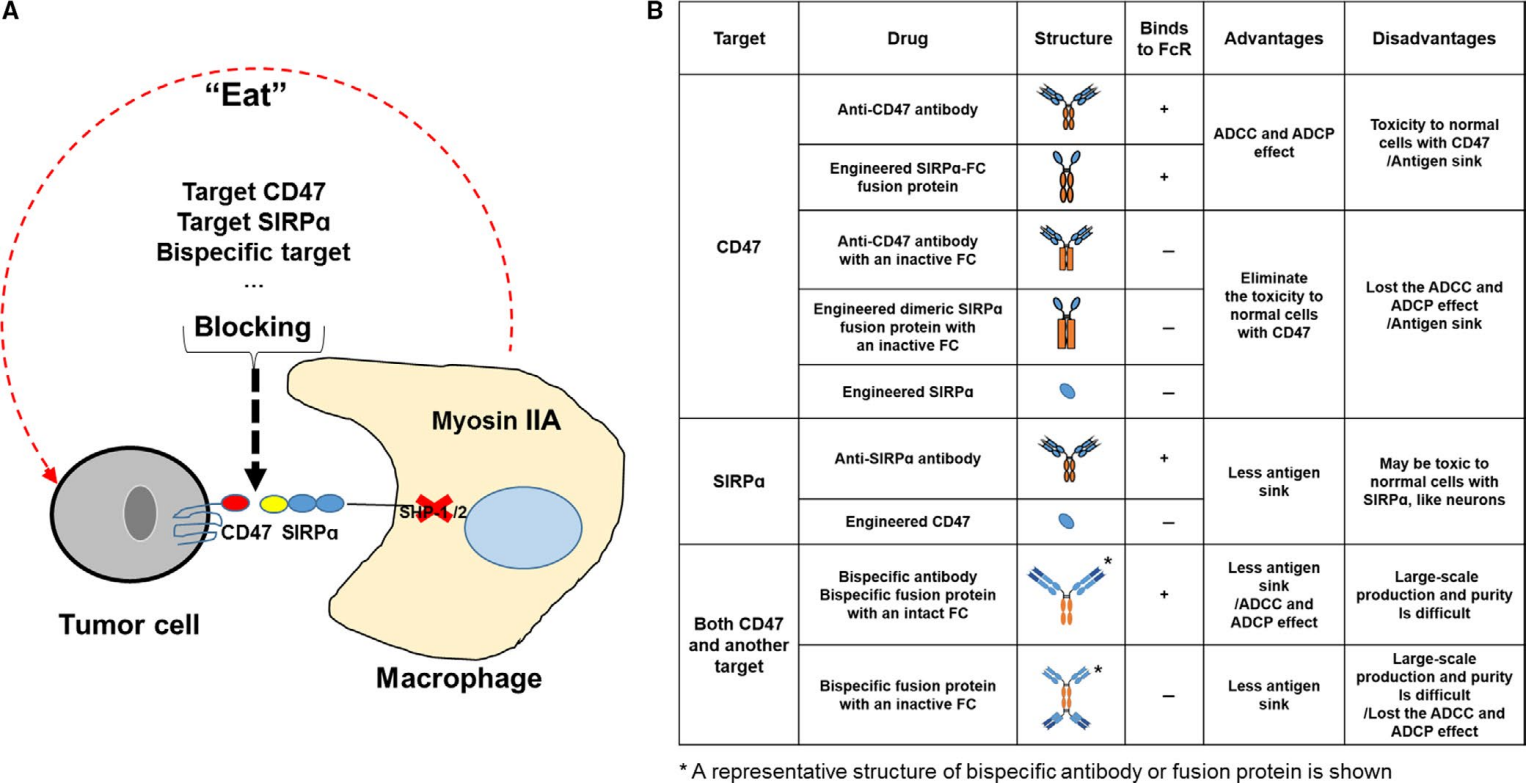
The traditional strategies for the treatment of cancers through the blockage of the CD47/SIRPɑ axis. (A) Antibodies or engineered proteins against CD47 or SIRPɑ are used to block the interaction between the 2 molecules. (B) The list of traditional strategies. The agents containing an intact Fc domain block the CD47/SIRPɑ interaction and induce antibody‐dependent cellular cytotoxicity (ADCC) or antibody‐dependent cellular phagocytosis (ADCP). Moreover, the agents containing an inactive Fc domain can eliminate the potential toxicity in normal cells with CD47, particularly normal red blood cells (RBCs). Usually, the monomeric agonists or the agents with an inactive Fc domain require synergy with tumor‐targeting antibodies to achieve improved anti‐cancer efficacy

The CD47/SIRPɑ axis is important for tumor progression. Therefore, blockage of this axis may be an effective treatment against hematologic and solid malignancies. Several molecules have been developed for the blockage of the CD47/SIRPɑ axis. Notably, some of these molecules, including CD47 targeting agents,^28^, ^29^, ^30^, ^31^, ^32^, ^33^ SIRPɑ targeting agents,^34^, ^35^ and bispecific target agents^18^, ^36^, ^37^, ^38^ are currently undergoing preclinical and clinical evaluation (Figure 3 and Table S1). All these agents are characterized by advantages and disadvantages regarding efficacy, toxicity, and other properties.

Among the aforementioned approaches, blockage of the CD47/SIRPɑ axis through the use of anti‐CD47 antibodies and CD47‐targeting recombinant proteins, is the leading therapeutic approach in both animal experimental models and clinical trials (Figure 3B). Numerous studies demonstrated that anti‐CD47 antibodies suppress tumor growth in the NSG (NOD‐SCID IL2Rgamma null) xenograft model, including non‐Hodgkin lymphoma, acute lymphoblastic leukemia, AML, and myeloma.^11^, ^13^, ^39^, ^40^, ^41^ In addition, therapies based on the use of an anti‐CD47 antibody are currently being investigated in clinical trials for the treatment of hematologic and solid malignancies (Table 1). For example, Hu5F9‐G4—a humanized antibody that contains a human IgG4 Fc fragment—is the first approved anti‐CD47 antibody for use in clinical trials to treat a spectrum of hematologic malignancies and solid tumors.^28^, ^42^ Recent clinical data of the combination of Hu5F9‐G4 and rituximab—an antibody against CD20– for the treatment of relapse or refractory non‐Hodgkin lymphoma showed an objective response rate of approximately 50%.^28^ In 2015 and 2016, a phase 1 clinical trial was initiated to investigate the use of the anti‐CD47 antibody CC‐90002 for the treatment of hematologic neoplasms.^29^ In 2018, a clinical trial was initiated to determine the usefulness of SRF231—another anti‐CD47 antibody—for the treatment of advanced solid and hematologic cancers.^32^

**Table 1:** The clinical trial data of CD47/SIRPɑ

**Drug**	**Target**	**Cured disease**	**Strategy**	**Start date**	**Sponsor**	**Phase**	**NCT number**
AO‐176	CD47	Solid tumor	Single agent	February, 2019	Arch oncology	Phase 1	NCT03834948
IBI187	CD47	Advanced malignancies	Single agent	January, 2019	Innovent biologics (Suzhou) Co. Ltd.	Phase 1	NCT03763149
IBI188	CD47	Advanced malignancies	Single agent or combination with Rituximab	December, 2018	Innovent Biologics (Suzhou) Co. Ltd.	Phase 1	NCT03717103
SRF231	CD47	Advanced solid cancers Hematologic cancers	Single agent	March, 2018	Surface oncology	Phase 1	NCT03512340
Hu5F9‐G4	CD47	Solid tumors	Single agent	August, 2014	Forty seven, Inc	Phase 1	NCT02216409
Hu5F9‐G4	CD47	Acute myeloid leukemia Myelodysplastic syndrome	Single agent	November, 2015	Forty seven, Inc	Phase 1	NCT02678338
Hu5F9‐G4	CD47	Lymphoma, non Hodgkin Lymphoma, large B‐Cell, diffuse Indolent lymphoma	Single agent combined with Rituximab	November, 2016	Forty seven, Inc The leukemia and lymphoma society	Phase 1/2	NCT02953509
Hu5F9‐G4	CD47	Colorectal neoplasms Solid tumors	Single agent combined with Cetuximab	November, 2016	Forty seven, Inc	Phase 1/2	NCT02953782
Hu5F9‐G4	CD47	Acute myeloid leukemia Myelodysplastic syndromes	Single agent or combined with Azacitidine	September, 2017	Forty seven, Inc	Phase 1	NCT03248479
TTI‐621	CD47	Hematologic malignancies Solid tumor	Single agent combined with Rituximab or Nivolumab	January, 2016	Trillium Therapeutics Inc	Phase 1	NCT02663518
TTI‐621	CD47	Solid tumors/breast/melanoma carcinoma	Single agent or combined with PD‐1/PD‐L1 inhibitor pegylated interferon‐#2a or T‐Vec or radiation therapy	September, 2016	Trillium Therapeutics Inc	Phase 1	NCT02890368
CC‐90002	CD47	Hematologic Neoplasms	Single agent or combined with Rituximab	March, 2015	Celgene corporation	Phase 1	NCT02367196
CC‐90002	CD47	Leukemia, myeloid, acute Myelodysplastic syndromes	Single agent	March, 2016	Celgene corporation	Phase 1	NCT02641002
ALX148	CD47	Metastatic cancer Solid tumor Advanced cancer Non Hodgkin lymphoma	Single agent combination with Pembrolizumab or Trastuzumab or Tuximab	February, 2017	Alexo therapeutics, Inc	Phase 1	NCT03013218
SIRPɑ Ab	SIRPɑ	Hepatocellular carcinoma	Collection of human samples	August, 2016	Nantes University Hospital	Investigation	NCT02868255

Targeting of CD47 through the use of recombinant proteins is another effective strategy in therapy based on the blockage of the CD47/SIRPɑ axis. TTI‐621—the first SIRPɑ‐Fc fusion protein—was produced by recombining the extracellular V‐set Ig domain of the wild‐type SIRPɑ with the human IgG1 Fc fragment, to compete with endogenous SIRPɑ in immune cells.^43^ In 2016, a phase 1 clinical trial was initiated to assess the usefulness of TTI‐621 against hematologic malignancies and solid tumors.^30^ In addition, CV1‐hIgG4 is an engineered protein with high affinity for CD47, generated by fusing a mutant V‐set Ig domain of SIRPɑ with the human IgG4 Fc scaffold. This engineered protein has shown great therapeutic efficacy in mice.^44^ Notably, CV1‐hIgG4 has shown obvious hematologic toxicity in mice and monkeys. Hence, ALX148 –a fusion protein with an improved safety profile—was constructed by combining CV1 to an inactive human IgG1 Fc domain. A phase 1 clinical trial was initiated to examine the usefulness of ALX148 for the treatment of solid tumors and non‐Hodgkin lymphoma.^33^

As mentioned earlier in this article, all the agents targeting CD47 are classified into two groups, namely one with an active Fc domain and another with an inactive Fc or absent Fc domain (Figure 3B). The former group of agents, including intact CD47 antibodies and recombinant proteins with an active Fc, blocks the CD47/SIRPɑ axis and initiates antibody opsonization. This process results in the destruction of the target cells through antibody‐dependent cellular cytotoxicity (ADCC) or antibody‐dependent cellular phagocytosis (ADCP).^39^, ^44^, ^45^, ^46^ However, due to the wide expression of CD47 in normal cells, agents targeting CD47 undergo the ‘antigen sink’ effect in vivo. Therefore, use of those with an active Fc domain may lead to the occurrence of various hematologic adverse effects, especially RBC toxicity.^14^, ^44^, ^47^ On the contrary, the toxicity to normal cells with CD47 induced by the latter group of agents, may be eliminated. However, the anti‐tumor efficacy may also be compromised, owing to the loss of the ADCC and ADCP effect.

Other strategies for the blockage of the CD47/SIRPɑ axis include the use of SIRPɑ‐targeting agents, and bispecific agents targeting CD47 and another molecule (Figure 3B and Table S1). Of note, attempts have been made to engineer CD47 proteins targeting SIRPɑ and anti‐SIRPɑ antibodies. Velcro‐CD47 (an engineered CD47 with high affinity for human SIRPɑ) synergizes with trastuzumab (an antibody against human epidermal growth factor receptor 2) or cetuximab (an antibody against epidermal growth factor receptor) to enhance the phagocytosis of SKBR3 breast cancer cells or DLD‐1 colon cancer cells, respectively.^48^ In a human SIRPɑ knock‐in mouse model, the anti‐SIRPɑ antibody KWAR23 synergizes with rituximab (an antibody against CD20) to inhibit the growth of human Burkitt lymphoma.^35^ Bispecific agents, simultaneously targeting CD47 and another tumor antigen, have also been developed to reduce the on‐target‐off‐tumor effect. These agents include bispecific antibodies and fusion proteins.^18^, ^37^, ^38^, ^49^, ^50^ For example, the bispecific antibody NI‐1701 targeting CD47 and CD19 showed a synergistic effect with rituximab in inhibiting the growth of human lymphoma cells in an NSG mouse model.^50^ Moreover, as a representative of dual‐targeting fusion protein, IAB targets both CD47 and PD‐L1. In addition to the common effects associated with the blockage of the CD47/SIRPɑ axis, IAB activates T cells and successfully suppressed the growth of MC38 in immune‐competent mice.^37^ Similar to the agents targeting CD47, those targeting SIRPɑ or the bispecific agents can be designed to contain an active Fc, aimed at eliciting the ADCC and ADCP effect. Unlike the CD47‐targeting agents, those targeting SIRPɑ avoid the “antigen sink” effect to a large extent, owing to the restricted expression pattern of SIRPɑ. However, the neurons expressing SIRPɑ may continue to be affected by agents targeting SIRPɑ.^5^ Bispecific agents designed to simultaneously target CD47 and other tumor antigens may minimize the development of adverse effects. However, the purity and mass production of such agents is currently challenging.^51^

Currently, the use of immune checkpoint inhibitors and CAR‐T therapy are the most successful approaches in the field of cancer immunotherapy. However, there is a lack of studies directly comparing the blockage of the CD47/SIRPɑ axis using these therapies. Since the syngeneic B16F10 melanoma mouse model is the most widely used model for the assessment of cancer immunotherapy, we would try to discuss about the efficacy of different approaches in this model. A study involving the use of a mouse CD47‐targeting nanobody A4 or anti–PD‐L1 antibodies in this B16F10 tumor syngeneic model did not show a delay in B16 growth or prolongation of mouse survival.^52^ However, the combination of the nanobody A4 and anti–PD‐L1 antibody significantly inhibited tumor growth and prolonged survival.^52^ In another study, monotherapy with an anti‐CTLA‐4 antibody significantly prolonged mouse survival. In contrast, the effectiveness of the nanobody A4 was inferior to that reported for the anti‐CTLA‐4 antibody, and did not show a synergic effect with anti‐CTLA‐4 therapy.^53^ In the same model, CAR‐T therapy using the novel nanobody A12 cells to target mouse PD‐L1 retarded the growth of WT‐B16 and B16 PD‐L1^hi^, and prolonged the survival of tumor‐bearing mice.^54^ In addition, the use of murine interleukin‐18‐secreting CD19 CAR‐T cells delayed the growth of B16 CD19 tumors.^55^

In conclusion, according to the currently available evidence, blocking the CD47/SIRPɑ axis or targeting PD‐L1 may not be sufficient for the effective treatment of B16F10 syngeneic tumors. In contrast, use of the anti‐CTLA4 antibody may exert an improved effect. Combination therapy of anti‐CD47 with other immune checkpoint‐targeting approaches may achieve an improved anti‐tumor effect. Additionally, CAR‐T‐based therapies appear to be more effective vs the aforementioned molecule‐based therapies. Nevertheless, the lack of studies directly comparing these therapies and absence of additional animal models, may compromise the validity of these conclusions. We suggest that different patients may exhibit varied sensitivity to different immunotherapy approaches. Hence, it is challenging to assert which approach offers superior results in this setting.

## 5 ENGINEERING MACROPHAGES FOR THE TREATMENT OF CANCER

**Figure 4:**
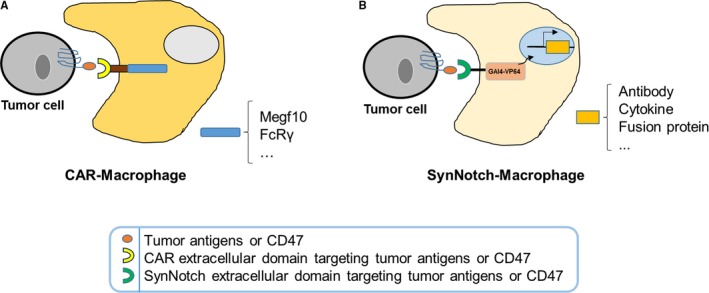
The proposed strategies for the engineering of macrophages against cancers. (A) Engineered macrophages with chimeric antigen receptors (CARs) for the phagocytosis of cancer cells. A proposed CAR macrophage with a CAR containing an extracellular scFv against a tumor antigen (or anti‐CD47 scFv, or an engineered extracellular domain of SIRPɑ) and an intracellular domain (Megf10 or FcRγ) can recognize and phagocytose cancer cells. (B) Engineered macrophages using the SynNotch system for the killing of cancer cells. Proposed SynNotch‐Macrophages containing a SynNotch receptor, which can recognize a tumor antigen (or CD47) and activate a spectrum of downstream genes, are able to eliminate cancer cells through multiple mechanisms. Among others, these mechanisms include the activation of genes encoding antibodies or engineered proteins to block the CD47/SIRPɑ axis and genes encoding variant cytokines to reverse the immune‐inhibitory microenvironment in tumors

In recent years, the combination of synthetic biology and cell therapy has led to a revolution in cancer immunotherapy, exemplified by the invention and successful clinical application of CAR‐T cells.^56^ Considering the important role of macrophages in the blockage of the CD47/SIRPɑ axis, it is rational to suggest that engineering macrophages to target CD47 may offer promising therapeutic efficacy. To this end, we propose a concept for the engineering of macrophages that can sense specific tumor antigens (including CD47), and respond through the activation of phagocytosis and secretion of therapeutic antibodies and cytokines (Figure 4).

Similar to CAR‐T cells, Chimeric Antigen Receptors for Phagocytosis (CAR‐Ps)‐macrophages have been designed to engulf specific targets.^57^ It has been reported that CAR‐Ps‐macrophages with the Megf10 or FcRγ intracellular region can specifically consume antigen‐coated synthetic particles and human cancer cells.^57^ We propose that blockage of the CD47/SIRPɑ axis using macrophages with CAR targeting CD47 may be an effective anti‐tumor therapy (Figure 4A). CD47‐CAR‐macrophages block the CD47/SIRPɑ axis and also self‐activate to attack the CD47‐positive cancer cells. The key to engineering an effective CAR‐Macrophage is to design an intracellular domain that can potently and enduringly activate macrophages. Although the optimization of the intracellular domain may be challenging, it may also be rewarding.

Apart from CAR‐Macrophages, other engineered macrophages may be developed for the treatment of cancers. In 2016, Lim et al invented the synthetic Notch (SynNotch) receptor, which consists of an extracellular ligand‐recognition domain, a notch core domain, and an intracellular transcriptional activation domain.^58^ Ligand recognition leads to cleavage of the notch core domain and release of the transcriptional activation domain. The latter translocates to the nucleus and subsequently initiates the expression of the target genes. Lim and his colleagues invented this system and used it to engineer T cells for the killing of cancer cells.^59^, ^60^ Solid tumors are infiltrated by more macrophages than T cells.^61^, ^62^ Therefore, given the successful application of the SynNotch system in T cells, we propose that macrophages engineered using this system may become a powerful approach in the treatment of cancers. The patient‐derived macrophages can be armed with the SynNotch receptor that targets the tumor antigen and elicits a customized response, such as the secretion of the PD‐L1‐blocking antibody (Figure 4B).

## 6 CONCLUSION

In the past, scientists struggled to employ macrophages for the treatment of cancer, with limited efficacy. Currently, the importance of the CD47/SIRPɑ axis offers new promise toward this goal. Preclinical studies demonstrated that activation of macrophages through blockage of the CD47/SIRPɑ axis may effectively treat various cancers. The currently ongoing clinical trials investigating therapies based on the blockage of the CD47/SIRPɑ axis will further determine its therapeutic efficacy in patients. In addition, the successful application of synthetic gene engineering in T cells revealed its versatile application potential. Therefore, utilizing a synthetic gene circuit to engineer macrophages may be a novel strategy for the treatment of cancer. Synthetic receptors and the CD47/SIRPɑ axis provide an opportunity to bring the potential value of macrophages in cancer immunotherapy into play.

## CONFLICTS OF INTEREST

The authors have no conflict of interest to declare.

## Data Availability

Data sharing is not applicable to this article as no new data were created or analyzed in this study.
