# Supplementary material for: Engineering macrophages to phagocytose cancer cells by blocking the CD47/SIRPɑ axis
Source: Cancer Med. 2019 Jun 11;8(9):4245–53. doi: 10.1002/cam4.2332 (PMC6675709; doi:10.1002/cam4.2332)
Supplement: Supplementary file 1 [file CAM4-8-4245-s001.pptx]

## Slide 1
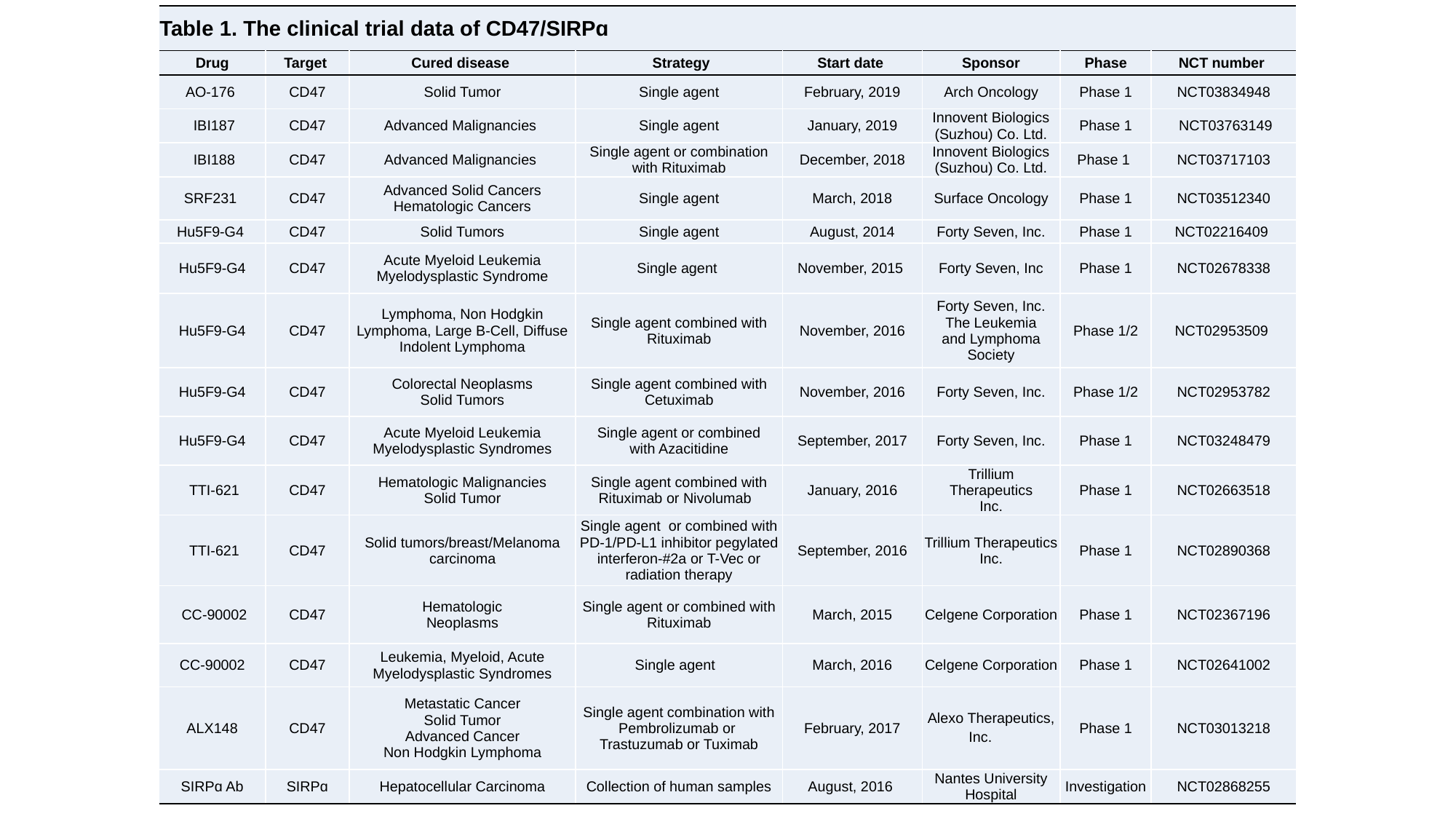

| Table 1. The clinical trial data of CD47/SIRPɑ | | | | | | | |
| --- | --- | --- | --- | --- | --- | --- | --- |
| Drug | Target | Cured disease | Strategy | Start date | Sponsor | Phase | NCT number |
| AO-176 | CD47 | Solid Tumor | Single agent | February, 2019 | Arch Oncology | Phase 1 | NCT03834948 |
| IBI187 | CD47 | Advanced Malignancies | Single agent | January, 2019 | Innovent Biologics (Suzhou) Co. Ltd. | Phase 1 | NCT03763149 |
| IBI188 | CD47 | Advanced Malignancies | Single agent or combination with Rituximab | December, 2018 | Innovent Biologics (Suzhou) Co. Ltd. | Phase 1 | NCT03717103 |
| SRF231 | CD47 | Advanced Solid Cancers Hematologic Cancers | Single agent | March, 2018 | Surface Oncology | Phase 1 | NCT03512340 |
| Hu5F9-G4 | CD47 | Solid Tumors | Single agent | August, 2014 | Forty Seven, Inc. | Phase 1 | NCT02216409 |
| Hu5F9-G4 | CD47 | Acute Myeloid Leukemia Myelodysplastic Syndrome | Single agent | November, 2015 | Forty Seven, Inc | Phase 1 | NCT02678338 |
| Hu5F9-G4 | CD47 | Lymphoma, Non Hodgkin Lymphoma, Large B-Cell, Diffuse Indolent Lymphoma | Single agent combined with Rituximab | November, 2016 | Forty Seven, Inc. The Leukemia and Lymphoma Society | Phase 1/2 | NCT02953509 |
| Hu5F9-G4 | CD47 | Colorectal Neoplasms Solid Tumors | Single agent combined with Cetuximab | November, 2016 | Forty Seven, Inc. | Phase 1/2 | NCT02953782 |
| Hu5F9-G4 | CD47 | Acute Myeloid Leukemia Myelodysplastic Syndromes | Single agent or combined with Azacitidine | September, 2017 | Forty Seven, Inc. | Phase 1 | NCT03248479 |
| TTI-621 | CD47 | Hematologic Malignancies Solid Tumor | Single agent combined with Rituximab or Nivolumab | January, 2016 | Trillium Therapeutics Inc. | Phase 1 | NCT02663518 |
| TTI-621 | CD47 | Solid tumors/breast/Melanoma carcinoma | Single agent or combined with PD-1/PD-L1 inhibitor pegylated interferon-#2a or T-Vec or radiation therapy | September, 2016 | Trillium Therapeutics Inc. | Phase 1 | NCT02890368 |
| CC-90002 | CD47 | Hematologic Neoplasms | Single agent or combined with Rituximab | March, 2015 | Celgene Corporation | Phase 1 | NCT02367196 |
| CC-90002 | CD47 | Leukemia, Myeloid, Acute Myelodysplastic Syndromes | Single agent | March, 2016 | Celgene Corporation | Phase 1 | NCT02641002 |
| ALX148 | CD47 | Metastatic Cancer Solid Tumor Advanced Cancer Non Hodgkin Lymphoma | Single agent combination with Pembrolizumab or Trastuzumab or Tuximab | February, 2017 | Alexo Therapeutics, Inc. | Phase 1 | NCT03013218 |
| SIRPɑ Ab | SIRPɑ | Hepatocellular Carcinoma | Collection of human samples | August, 2016 | Nantes University Hospital | Investigation | NCT02868255 |

## Slide 2
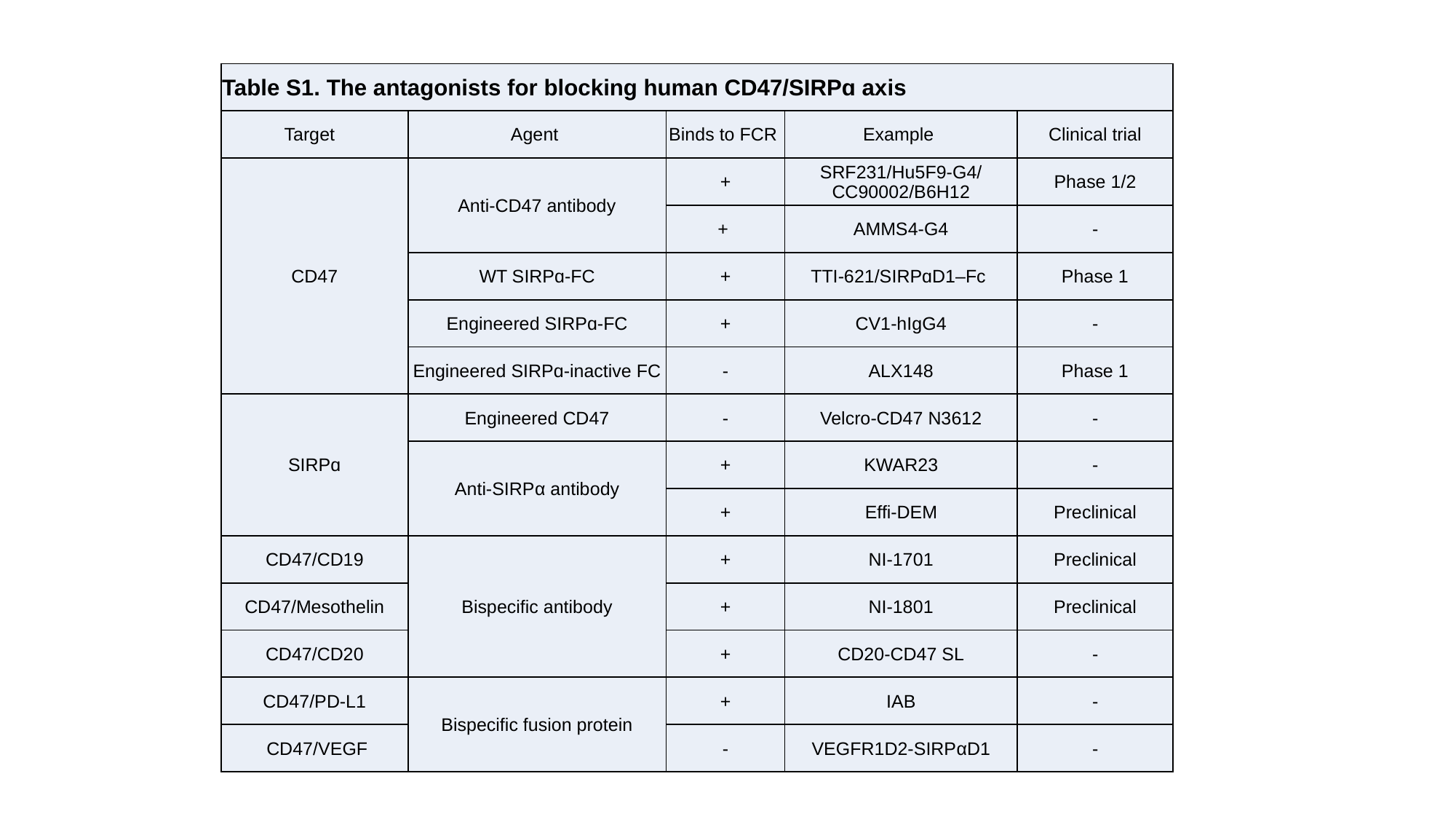

| Table S1. The antagonists for blocking human CD47/SIRPɑ axis | | | | |
| --- | --- | --- | --- | --- |
| Target | Agent | Binds to FCR | Example | Clinical trial |
| CD47 | Anti-CD47 antibody | + | SRF231/Hu5F9-G4/CC90002/B6H12 | Phase 1/2 |
| | | + | AMMS4-G4 | - |
| | WT SIRPɑ-FC | + | TTI-621/SIRPɑD1–Fc | Phase 1 |
| | Engineered SIRPɑ-FC | + | CV1-hIgG4 | - |
| | Engineered SIRPɑ-inactive FC | - | ALX148 | Phase 1 |
| SIRPɑ | Engineered CD47 | - | Velcro-CD47 N3612 | - |
| | Anti-SIRPα antibody | + | KWAR23 | - |
| | | + | Effi-DEM | Preclinical |
| CD47/CD19 | Bispecific antibody | + | NI-1701 | Preclinical |
| CD47/Mesothelin | | + | NI-1801 | Preclinical |
| CD47/CD20 | | + | CD20-CD47 SL | - |
| CD47/PD-L1 | Bispecific fusion protein | + | IAB | - |
| CD47/VEGF | | - | VEGFR1D2-SIRPαD1 | - |
